# Measuring the unmeasurable: defining and rating precarity with the aid of EU-LFS data

**DOI:** 10.1007/s43545-023-00651-5

**Published:** 2023-03-22

**Authors:** Maria Symeonaki, Glykeria Stamatopoulou, Dimitrios Parsanoglou

**Affiliations:** Department of Social Policy, School of Political Sciences, Panteion University of Social and Political Sciences, 136 Syggrou Av., 17671 Athens, Greece

**Keywords:** Precarity, Precarious labour, Type of contract, Entitlements, Job insecurity

## Abstract

Precarity has been established as a central theoretical issue in labour market research and numerous attempts have been made in the past to provide indicators that measure it. Precarity has also been present in political discourse and linked to specific new forms of employment (temporary, part-time, insecure, and atypical amongst others) and certain social groups often defined as vulnerable groups (youth, women, ethnic minorities). However, precarity still remains a phenomenon that needs to be quantified with the use of reliable data. The present paper aims at providing a methodology for measuring individuals that are in precarious employment with data drawn from the EU-Labour Force Survey (EU-LFS). Thus, it presents a way of identifying individuals in the core of precarity and others that belong to this set to a lesser degree. More specifically, four different levels of precarity are identified and the methodology is illustrated and tested for a specific case study, that of Greece. However, the proposed technique can be applied with no or minor modifications to other data sets of EU member states, where the common EU-LFS questionnaire is used. An effort is also made to recognise the socio-demographic characteristics of the individuals that are identified as being precarious belonging to the four levels of precarity and to specify their differences. The analysis yields that as we move from the fist level of weak precarity to the last one of strong precarity the individuals become younger, worse paid and better educated.

**Supplementary Information:**

The online version contains supplementary material available at 10.1007/s43545-023-00651-5.

## Introduction

Despite the rising usage of the term precarious employment, it does not seem to have a universally accepted definition across Europe (Eurofound 2018). As stated later in Rönnblad et al. (2019) “a clear multi-dimensional definition of precarity employment is lacking, and harmonization efforts are needed”. Bodin et al. (2021) also highlight the need of a consensus on a definition of the term precarious employment and in a similar vein Kreshpaj et al. (2021) explain how “the lack of a common definition for precarious employment severely hampers the comparison of studies within and between countries, consequently reducing the applicability of research findings”. However, regardless of the absence of an agreed definition, the conceptualisation of precarity has become nowadays a significant matter at the European level. Recently, a relevant survey was conducted for the first time in 2022, namely the European Barometer of Precariousness and Poverty Survey, to observe the social situation, as well as the opinions and concerns of the inhabitants of six European countries (i.e., France, United Kingdom, Germany, Greece, Italy and Poland) (Mercier et al. 2022). Six thousand Europeans were asked, and the results show that 27% of the respondents believe that they are in a precarious situation, where an unexpected expense could push them into poverty. The respective percentage for Greece is 51%. Amongst the different categories of populations perceived as potentially precarious are the elderly with the exception of Greece and Italy where young people are identified as more vulnerable. According to the same source, 55% of Europeans feel that there is a significant risk that they will find themselves in an unstable financial situation in the coming months. In addition, 39% state that they have accepted a job they didn’t want (11% of the respondents say this has happened during the last six months). The latest results from EUROSTAT on precarious employment (measured only as the percentage of employees with a short-term contract of up to 3 months) show that it was equal to 2.4% in the EU-27 in 2019.

In July 2017, the European Parliament accepted a resolution on working conditions and precarious employment. The resolution defined precarious employment as “employment which does not comply with EU, international and national standards and laws and/or does not provide sufficient resources for a decent life or adequate social protection”. Precarious employment and precarity have from the start been linked to the reign (or the ‘terror’) of neoliberalism (Giroux 2005). More precisely, the discourse on precarious labour was strongly associated with the discourse on labour market flexibility, which emerged from the 1980s onwards (Atkinson and Meager 1986; Klau and Mittelstadt 1985; Reilly 2001; Rodgers 2007). Under this scope, precarity came to describe “the labour conditions that arose after the transition from life-long, stable jobs common in industrial capitalist and welfare-state economies, to temporary, insecure, low-paying jobs emerging with the globalization of the service and financial economy” (Casas-Cortes and Cobarrubias 2007: 115). In the same spirit, Guy Standing (2011: 1) arrives to the point where he claims that “the result [of labour market flexibility] has been the creation of a global *precariat*, consisting of many millions around the world without an anchor of stability. They are becoming a new dangerous class”. The discourse on precarity has later on been developed to encompass all possible shapes of unsure, not guaranteed, flexible types of work: from illegalised, seasonal and temporary employment to homework, flex- and temp-work to subcontractors, freelancers or so-called self-employed persons. In other words, discourses on precarity appeared as a critical counterweight to the optimistic assessments of the flexible post-Fordist labour and welfare regimes. Whilst discourses on labour market flexibility, both in their extreme or moderate versions, insisted on the economic optimisation that flexible working arrangements could generate within an increasingly competitive globalised environment, discourses on precarity highlighted the degrading effects of the *flexibilisation* or *casualisation* of labour markets on labour protection laws and on multiple benefits guaranteed by the previous welfare-state economy (Casas-Cortes and Cobarrubias 2007).

The main theoretical foundations of the precarity discourse(s) can be detected within two main schools of thought: on the one hand, the post-Marxist thinking of the Italian autonomy tradition blended with the Foucauldian tradition of biopolitics (Gill and Pratt 2008); on the other hand, the accounts of the regulation school (Aglietta 1979; Bonefeld and Holloway 1991; Lipietz 1992), which produced the central critical concepts for the socio-economic analyses of the post-Fordist regimes of accumulation and labour relations. In this context and in more precise terms, precarity has encompassed unequally two shifts that largely shaped the post-industrial societies (Bell 1973; Touraine 1969): on the one hand, the new creative industries that emerged from the micro-informatics and ICT revolutions and, on the other hand, the unprecedented expansion of the tertiary sector of the economy that has been including more and more disparate types of services.

What renders precarious labour a paradigmatic form of labour in the post-Fordist era is not only its flexible character, nor its allegedly cognitive content and requirements. Contemporary sociological research and critical political thought have defined precarious labour as the multiplication of non-standard and irregular (not in the strict legal sense) forms of labour (Mezzadra and Neilson 2013), bearing the following characteristics (Papadopoulos et al. 2008):

1. They are contract-based, part-time or of definite duration.
2. They are organised around the product of work—whether in the form of subcontracting, project-specific or freelance work—where remuneration depends on the quality of the product, e.g., in the creative industries or in research.
3. They are organised beyond the existing welfare structures, e.g., social insurance, unemployment, and other benefits like motherhood etc.
4. They are characterised by increasing mobility, global, regional but also within national borders.
5. They are encouraging and even imposing intersectoral mobility of workers.
6. They are covering a wide range of unequally remunerated workers, from working poor to highly paid temporary skilled workers, and
7. They are lacking unionism despite sporadic efforts for a connection with traditional unions, like the case of the *intermittents du spectacle* in France or the platform workers in several countries such as Italy, Germany, and recently Greece.

Precarity as a descriptive notion meets widely expanded realities particularly in some social contexts, interestingly enough not only in those affected by the 2008 financial and economic crisis since precarious labour is not at all necessarily linked to high unemployment rates; equally if not more importantly, precarity as a concept of political sociology can provide useful insights as for the agency of precarious workers. Following Judith Butler’s (2004) distinction between “precarity” (intended in the labour market sense) and “precariousness” (as an ontological and existential category that describes the common, but unevenly distributed, fragility of human corporeal existence), the precarious subject has been forged both by academic and activist idioms as the potential tool to crack new codes of labour subjectivities that cannot be fit into existing forms of collective agency and representativity (Neilson and Rossiter 2005).

However, it seems that the experiences and the actions of precarious workers cannot be seen as having an analogy with earlier forms of collective identities. Precarious labour exists only in the plural, as a multiplicity of experiences variously positioned, exploited, and lived within contemporary capitalism, and not as a unified subjectivity or a ‘precariat’ which forms a distinct social subject (Trimikliniotis et al. 2016). It also seems that precarity can entail diverse and contradictory realities and experiences; precarity is in fact an uncertain but also multidimensional condition: “We are precarias. This means some good things (such as the accumulation of knowledge, expertise and skills through our work and existential experiences which are under permanent construction), a lot of bad ones (such as vulnerability, insecurity, poverty, social instability), and the majority, ambivalent stuff (mobility, flexibility)” (Precarias a la Deriva 2004: 17). Possibly this is the crucial point of the concept: it provides a theoretical and empirical basis of/for new forms of subjectivities and agencies that fundamentally form contemporary labour.

**Fig. 1:**
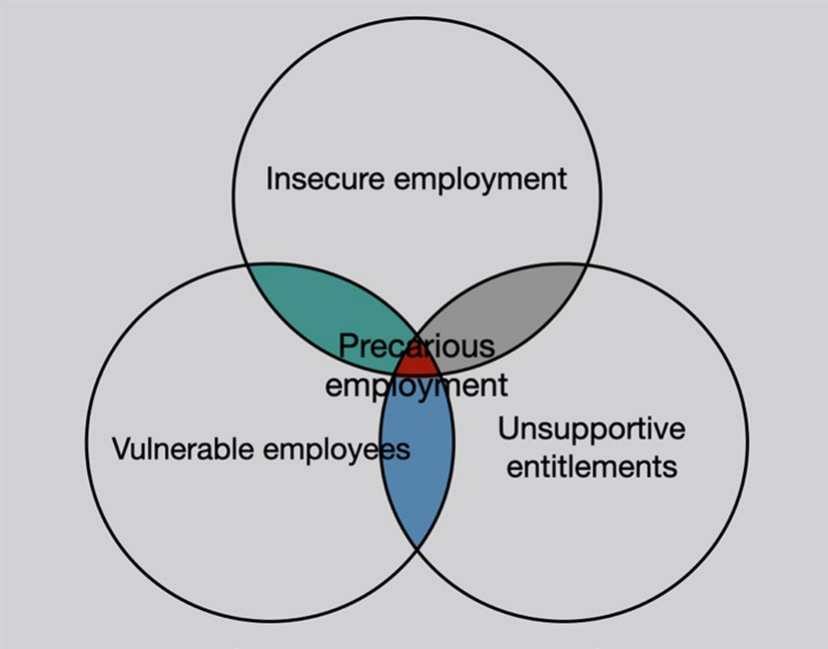
Precarious employment as the intersection of vulnerable employees, insecure employment, and unsupportive entitlements (adapted from Olsthoorn 2014)

Motivated by the theoretical significance that precarity has been gaining during the last decades, the paper provides a set theory methodology for measuring individuals in precarious employment using raw data drawn from the EU-LFS, a very relevant database for examining labour market conditions in Europe. The main impetus behind the study is therefore to provide a way to measure precarious employment that can be used for comparisons within and amongst European countries. The EU-LFS is specifically designed for collecting information concerning the labour force and is the largest sample survey of European households or individuals. Another database that could be used is the European Social Survey (ESS) that includes a number of variables that are relevant (Stamou et al. 2022b). However, the EU-LFS is preferable due to the increased sample size and relevance. More precisely, four different levels of precarity are provided starting from a level of weaker precarity up to the level of strong precarity. In order to quantify precarious workers, one would have to define first the specific characteristic(s) that would render someone precarious. Olsthoorn (2014: 424) conceptualises precarious employment as a characteristic of the employment relation, i.e., as insecure jobs occupied by vulnerable employees, who can expect few entitlements to income support when unemployed. In a very similar vein, Gunn et al. (2021) use as a definition that “precarious employment is conceptualized as a multi-dimensional construct including but not limited to employment insecurity, income inadequacy, and lack of rights and protection in the employment relation”. Moreover, a systematic review of definitions and operationalisations from quantitative and qualitative studies concerning precarious employment was conducted by Kreshpaj et al. (2020) using an initial set of 1225 studies which were narrowed down to 63 with the use of specific excluding criteria. The results of the qualitative thematic-analysis performed on these studies “resulted in a multidimensional construct including the following three dimensions: employment insecurity, income inadequacy, and lack of rights and protection”. Olsthoorn’s definition of precarious employment is fully compliant with these results. To express Olsthoorn’s definition using set theory, a branch of mathematical logic that studies *sets*, i.e., a collection of objects with certain properties, one would accept that precarious workers are those belonging to the intersection of insecure employment, vulnerable employees and unsupportive entitlements (Fig. 1). The reader can consult Appendix A for the basic concepts and notation of set theory.

However, each domain is associated with a number of variables and not only one, and each one of these variables increase the confidence that precarity is present to a lesser or greater extent. In the present paper we provide a set theory definition of precarity and quantify precarious workers using the EU-LFS data for the case of Greece. The proposed methodology can be used for all European countries participating in the EU-LFS with minor modifications that are sketched in Appendix B. More specifically, Sect. 2 describes the definitions and the methodology used to cover the different levels of precarity. Section 3 provides the results of the implementation to the Greek data and the socio-demographic characteristics of those identified as belonging to the suggested four levels of precarity. We explicitly seek to extract knowledge on whether precarity affects individuals differently according to their age, gender, educational level, occupation, marital status, area of living, occupation, and sector of economic activity. Several studies present the percentage differences in male and female (part-time) employment in European countries for different age categories (Burri and Aune 2014; Karamessini et al. 2016, 2019a, b; Plantenga et al. 2013). Symeonaki and Filopoulou (2017) provide a method for quantifying gender differences in occupation and education in European countries. More recent results provided by EUROSTAT for the year 2021, reveal gender differences in (involuntary) part-time employment. More precisely, amongst female part-time workers, more than 40% stated that they want to work more in three countries, namely Greece, Cyprus, and Spain. Particularly Greece exhibits the highest percentage equal to 54.4%. On the contrary, the percentage was less than 10% in Austria, Germany, and the Czech Republic. Rivero et al. (2021) highlight that women are more exposed to precarious employment and psychosocial risk factors than men. Oddo et al. (2021) introduced a precarity score based on longitudinal surveys in the USA and conclude that the precarity score was higher amongst women, people of colour and those less educated. Moreover, differentiations in respect to age and in relation to a fragile and uncertain career start are examined in the literature. However, an important strength of the proposed approach is the establishment of a method to select individuals across the precarity continuum and then thoroughly examine a sufficient number of socio-demographic characteristics to extract knowledge concerning the four proposed levels of precarity. Finally, Sect. 4 provides the conclusions of the study, discussion, and aspects of future work.

## Quantifying precarity with the use of EU-LFS data

Using Olsthoorn’s conceptualisation a precarious worker would have to be insecure, vulnerable and have limited entitlements. Each domain is linked with a number of variables some of which can be measured with the use of reliable data sources such as the EU-LFS. The EU-LFS is a cross-sectional household sample survey and the main source that provides monthly, quarterly, and annual data on the participation of Europeans in the labour market, their working conditions, and their job characteristics. At the same time, it is the basis for the estimation of unemployment rates and other structural indicators, such as inactivity or NEET rates, participation in life-long learning etc. by EUROSTAT. Therefore, the survey provides a way of measuring significant characteristics, whilst common classifications ensure the comparability between EU member countries. More information on the strengths and weaknesses of the EU-LFS are provided in Appendix B.

We first examine the variables linked with precarious employment in each of the domains earlier presented that are measured in the EU-LFS survey. In accordance with Olsthoorn (2014) three domains are considered: the Insecure employment domain (type of contract), the Unsupportive entitlements domain and the Insufficient resources domain. More specifically, concerning the Insecure employment domain (type of contract), employed individuals may be differentiated regarding their contract type. For a more complex, multidimensional index of early job insecurity, see Symeonaki et al. (2019), where the evolution of early job insecurity in European countries is examined. In this domain, we consider individuals having temporary employment (TE), part-time employment (PTE), involuntary part-time workers (IPT), involuntary temporary workers (ITE) and individuals whose temporary job/work contract is of limited duration and more specifically less than three months. More precisely, the EU-LFS asks respondents about:

- **Temporary employment (TE):** Compared to an open-ended permanent contract, a temporary employment is defined as a contract with a limited lifespan, the end of which “is determined by objective conditions such as reaching a certain date, completion of an assignment, or return of another employee who has been temporarily replaced” (OECD 2020).
- **Involuntary temporary employees (ITE):** In temporary employment the contract can end at a specific date. People considered as having temporary employment involuntarily are those who are engaged in temporary contracts because they could not find a permanent job. In the EU-LFS respondents are asked to choose the reason why they have a contract of limited duration. More specifically, the categories from which respondents can choose are the following: (1) person has a contract covering a period of training (apprentices, trainees, research assistants, etc.), (2) person could not find a permanent job, (3) person did not want a permanent job, (4) it is a contract of probationary period. Thus, involuntary employees correspond to the second category, defined as a percentage of total temporary employees.
- **Part-time employment (PT):** The variable in the EU-LFS is based on the respondents’ own view about their main job (self-assessment), and not on an objective measurement of the actual hours that they have worked per week.
- **Involuntary part-time employment (IPT):** People considered as working part-time involuntarily are those who want to have a full-time job but could not find one. More specifically, respondents are requested to elaborate on the reason why they are working part-time and choose from the following: (1) person is undergoing school education, or training, (2) of own-illness or disability, (3) looking after children or incapacitated adults (4) other family or personal reasons, (5) person could not find a full-time job. Involuntary part-time employees correspond to the last category and are defined as the percentage of total part-time employees.
- **Length of contract (< 3) (LT3):** The duration of the contract is specifically examined with predefined categories given to respondents (i.e., less than one month, 1 to 3 months, 4 to 6 months, …, more than 3 years). Therefore, one could filter out respondents that have a very short contract, less than three months. We note here that precarious employment is measured by EUROSTAT using solely this variable. We therefore use the 3-month length cut-off to follow EUROSTAT’s definition of precarious worker, i.e., someone whose job/work contract did not exceed three months duration.

In regard to the *Unsupportive entitlements domain* respondents are asked about the kind of health insurance they have and the type of their social security benefits. Therefore, respondents with no health insurance and/or no social security could be identified:

- **Lack of health insurance (WHI):** Respondents without any health insurance can be filtered out, i.e., those that select the predefined answer “no health insurance”, and
- **Lack of social security (WSS):** In a similar way, respondents without social security can also be recognised.

In the *Insufficient resources domain*, the following variables are of importance:

- **The total net monthly income:** This variable can be used to identify **low-paid workers**. Respondents recognised as low-paid would have monthly earnings less than the two-thirds of the median of monthly earnings. This is commonly used to examine domestic wage equity and social cohesion, and it is a measure for individual economic difficulties (UNECE 2015). It is also the OECD definition of low pay (OECD 2020), a cut-off point included in ILO’s global wage report 2020–21 (ILO 2020) and Ioakimoglou and Soumeli (2002).
- **Supplementary sources** of income apart from the main job are also examined. The participants surveyed when asked about their sources of income can choose from a predefined list of sources. Those are: (1) work, (2) age pension, (3) death pension, (4) disability pension, (5) income from movable or immovable property, (6) from other household members, (7) from people that do not belong to the household, and (8) benefits and allowances. Recoding this variable allows us to separate those that have other supplementary sources (categories 2–8) from those who do not have other sources than work (category 1). Supplementary sources, i.e., other sources of income, a notion clearly presented in Olsthoorn (2014) and Vosko (2006), relates to the issue of relative poverty and it allows for distinguishing different levels of precarity between workers, for example those that are low-paid with no supplementary income and those that are low-paid but have other sources of income.

We now define the following sets that correspond to the *Insecure employment* domain:

$$TE = { }\left\{ {{\text{x}}/{\text{x an individual who has a temporary job}}/{\text{work with limited contract}}} \right\}$$

$$\begin{gathered} ITE = { }\{ {\text{x}}/{\text{x an individual who has a temporary job}}/{\text{work with limited contract }} \hfill \\ {\text{because he}}/{\text{she could not find a permanent job}}\} { } \hfill \\ \end{gathered}$$

$$PT = { }\left\{ {{\text{x}}/{\text{x an individual who has a part}} - {\text{time job}}/{\text{work}}} \right\}$$

$$PT = { }\left\{ {{\text{x}}/{\text{x an individual who has a part}} - {\text{time job}}/{\text{work because he}}/{\text{ she could not find a full}} - {\text{time job}}} \right\}$$

$$\begin{gathered} LT3 = \{ {\text{x}}/{\text{x}} {\text{ an individual whose total duration }} \hfill \\ {\text{ or temporary work or work contract is less than three months}}\} \hfill \\ \end{gathered}$$

**Fig. 2:**
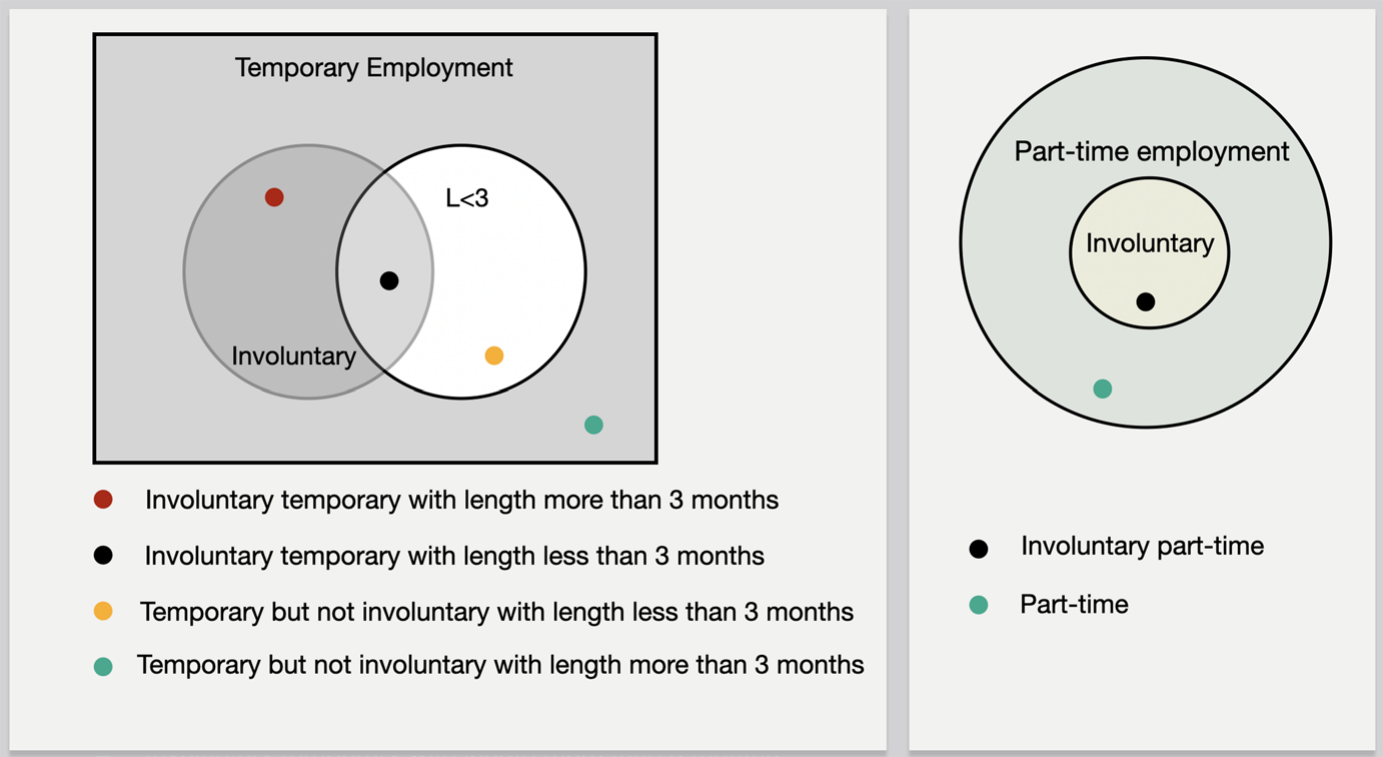
Venn diagrams of Temporary employment and Part-time employment

Figure 2 depicts the Venn diagrams of temporary employment and part-time employment. Involuntary temporary employment (ITE) is clearly a subset of Temporary employment (TE) (i.e., $ITE \subseteq TE$). Equality would hold in the extreme case where all temporary workers are involuntarily employed on a temporary contract. Moreover, LT3 is a subset of TE, since all workers with a contract lasting less than three months are temporarily employed. The three sets (TE, ITE and LT3) could have an intersection that would correspond to workers that are both part-time and temporary, with a contact lasting less than three months.

The different cases are exhibited in Fig. 2. In the case of a temporary contract, for example, an employed individual x could either:

$${\text{x}} \in TE{\text{ AND }} {\text{ x}} \notin ITE{\text{ AND }} {\text{ x}} \notin LT3$$

$${\text{x}} \in TE{\text{ AND }} {\text{ x}} \notin ITE {\text{ AND }} {\text{ x}} \in LT3$$

$${\text{x}} \in TE{\text{ AND }} {\text{ x}} \in ITE {\text{ AND }} {\text{ x}} \in LT3$$

$${\text{x}} \in TE {\text{ AND }} {\text{ x}} \in ITE {\text{ AND }} {\text{ x}} \notin LT3.$$

A part-time worker could either be working part-time involuntarily or with his/her own will:

$${\text{x}} \in PT {\text{ AND }} {\text{ x}} \notin IPT$$

$${\text{x}} \in PT{ }{\text{ AND }} {\text{ x}} \in IPT$$

**Fig. 3:**
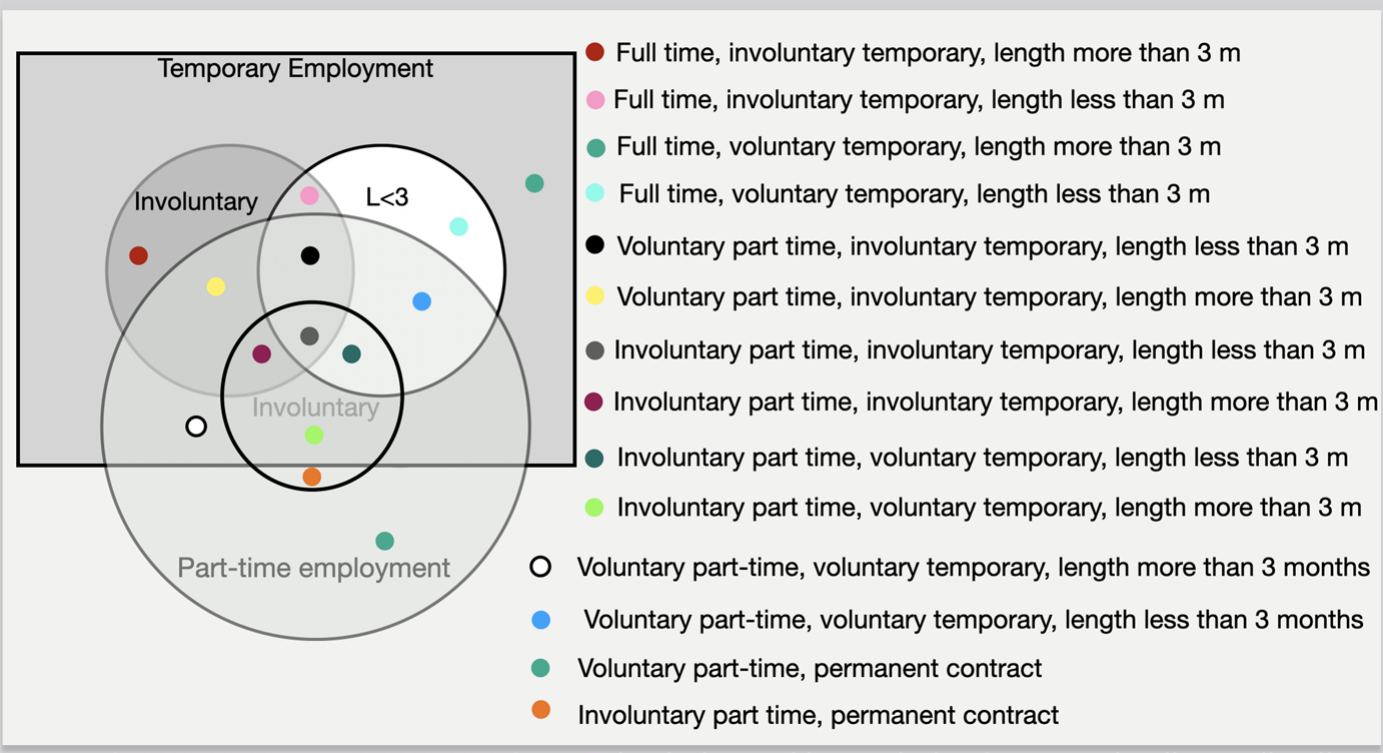
Venn diagram of Temporary and Part-time employment

Since the above conditions, i.e., that of being part-time or temporary employed are not mutually exclusive, to further examine the *Insecure employment* domain, one would have to look into the possible combinations of the realisation exhibited and explained in Fig. 3. Thus, when the quality of his/her job is examined focussing on the type of contract, an employed individual would have to be included in one of the categories presented in Fig. 3 and laid out exhaustively in Appendix C.

Apparently, these categories are associated with different levels of insecurity. To the one end, we could classify individuals with a permanent contract who are working voluntarily part-time as those having less insecurity and at the other end, those being involuntarily part-time and having against their will a temporary contract that lasts less than 3 months, as those being more insecure.

In respect with *the Unsupportive entitlements’ domain* individuals without social security and/or without health insurance are considered. The following sets are then defined:

$$SS = { }\left\{ {{\text{x}}/{\text{x an individual with no social security}}} \right\},\;{\text{and}}$$

$$WHI \; = \;\left\{ {{\text{x}}/{\text{x}}\;{\text{an individual with no health insurance}}} \right\}.$$

**Fig. 4:**
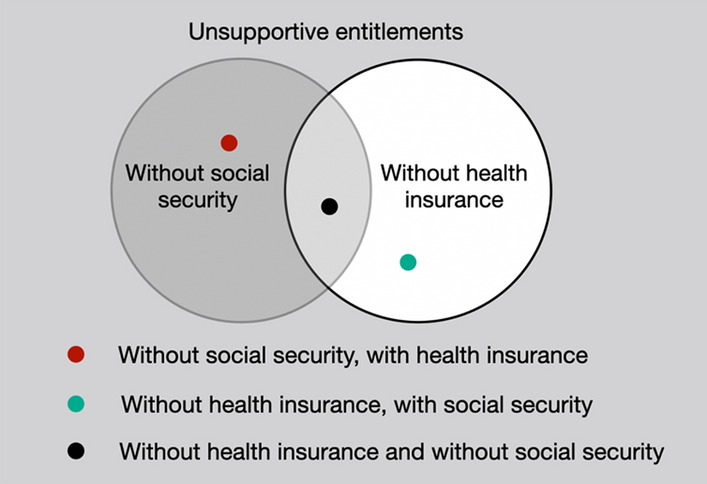
Venn diagram of Insecure Employment

In this sense, an individual with unsupportive entitlements (Fig. 4) would either:

$${\text{x}} \in WSS{\text{ AND }} {\text{ x}} \notin WHI$$

$${\text{x}} \notin WSS {\text{ AND }} {\text{ x}} \in WHI$$

$${\text{x}} \in WSS{\text{ AND }}{\text{ x}} \in WHI$$.

These categories are exhibited also in Appendix C.

In a similar way, different degrees of uncertainty are present here, with individuals with no social security and no health insurance being in the worse position comparatively.

We now define *the Insufficient resources domain*. This is defined by individuals having a poor income (less than the 2/3 of the median of the incomes reported) and/or no other sources than their work. Accordingly, the following sets are defined:

$$LP = { }\;\left\{ {{\text{x}}/{\text{x an individual with income}} < \frac{2}{3}{\text{median}}} \right\},\;{\text{and}}$$

$$WS = { }\left\{ {{\text{x}}/{\text{x an individual with no other sources}}} \right\}.$$

**Fig. 5:**
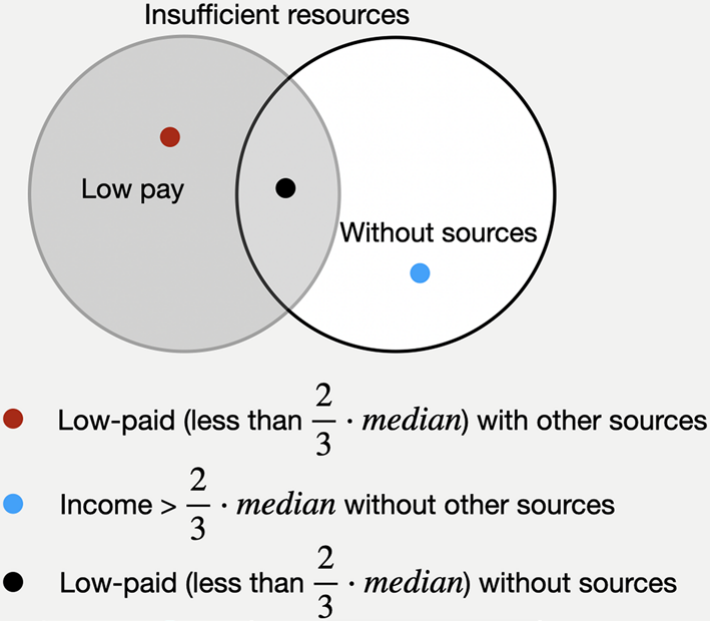
Venn diagram of Insufficient Resources domain

In this sense an individual has insufficient resources if he/she would belong to the categories presented in Fig. 5 and detailed in Appendix C:

$${\text{x}} \in LP {\text{ AND }} {\text{ x}} \notin WS$$

$${\text{x}} \notin LP {\text{ AND }} {\text{ x}} \in WS$$

$${\text{x}} \in LP {\text{ AND }} {\text{ x}} \in WS$$

In the most disadvantaged position in this domain are individuals that are low-paid and have no other sources than work.

**Fig. 6:**
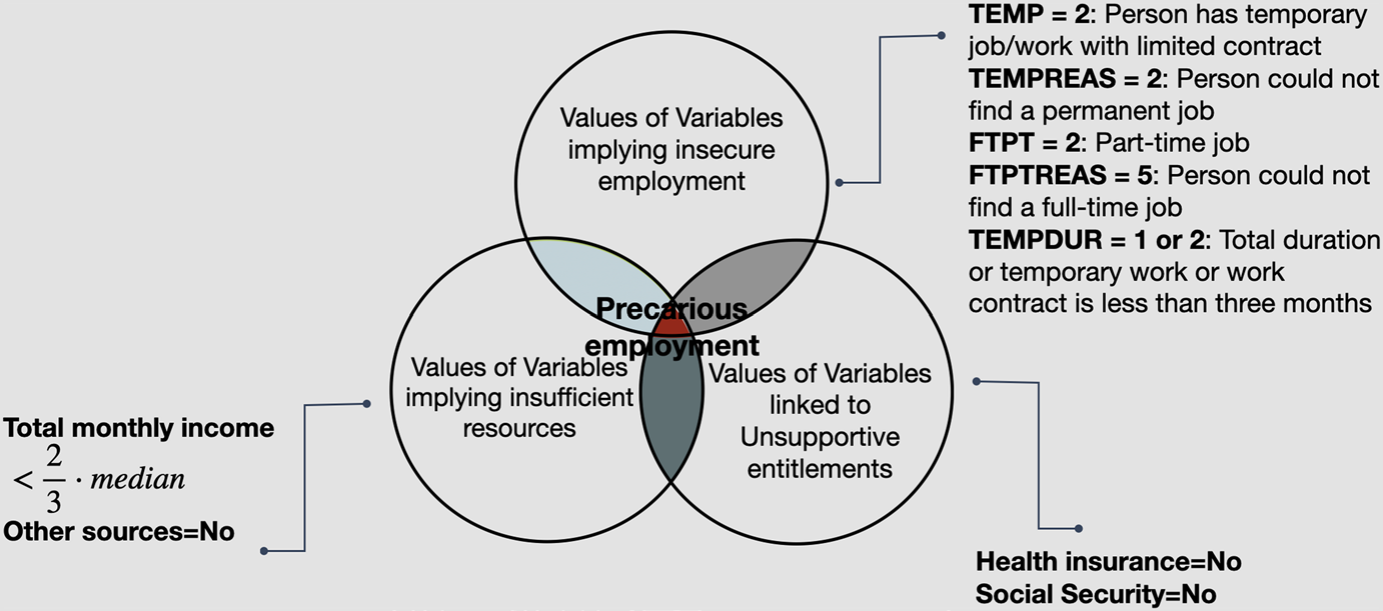
Venn diagram of precarious employment with the use of the EU-LFS data

Figure 6 explains the specific variables used in the EU-LFS and their respective values.

For the first level of precarity, that corresponds to weak precarity, we apply a more relaxed condition. More specifically, individuals belong to this category if they display at least one characteristic from each domain, i.e., *Insecure employment, Unsupportive entitlements*, and *Insufficient resources*. Individuals that are, for example, part-time employed with no social security and low income will belong to this category. This level of precarity, in contrast to the next three, considers part-time and temporary employment without restricting it to involuntary part-time and involuntary temporary contracts. All categories of part-time workers are regarded, either involuntary part-timers, or those giving other reasons for working part-time. However, they must exhibit at least one attribute from the other two domains, i.e., work without having social security and/or health insurance and be low-paid and/or with no supplementary sources of income. More specifically, using set theory notation we define that an individual ${\text{x}}$ belongs to the first level of precarity, if he/she belongs to:

- the union of all sets in Fig. 3 (Insecure employment), meaning that: ${\text{x}} \in TE {\text{ OR }} {\text{ x}}\in PT$ **AND**
- the union of all sets in Fig. 4 (Unsupportive entitlements), meaning that: ${\text{x}} \in WSS {\text{ OR }} {\text{ x}} \in WHI$, **AND**
- the union of all sets in Fig. 5 (Insufficient resources), meaning practically that: ${\text{x}} \in LP{\text{ OR }} {\text{ x}} \in WS$

More precisely:

### Definition 1:

We define as the set of *precarious workers – level 1 (weak precarity)* the set of individuals:

$$P_{1} = \{ {\text{x}}/{\text{x}}:\left\{ {{\text{x}} \in TE \  {\text{OR}} \  {\text{x}} \in PT\} {\text{ AND }} \{ {\text{x}} \in WSS {\text{ OR }} {\text{x}} \in WHI} \right\} {\text{ AND }} \left\{ {{\text{x}} \in LP {\text{ OR }} {\text{x}} \in WS} \right\}\}.$$

Thus, the members of this set are individuals that are either in part-time or temporary employment, and have no social security or medical insurance, with low earnings or with no other supplementary sources.

If the criteria become stricter, different levels of precarity could be identified. For example, replacing temporary and part-time employment with involuntary temporary and involuntary part-time employment the next level of precarity could be defined.

### Definition 2:

We define as the set of *precarious workers – level 2* the set of individuals:

The next level that includes even more precarious individuals, i.e., the set $P_{3}$, is determined using a stricter criterion and detects individuals that are involuntary temporary or involuntary part-time, without social security or health insurance, that are low-paid and moreover have no other sources apart from their work. Therefore:

$$P_{2} = \left\{ {{\text{x}}/{\text{x}}:\left\{ {{\text{x}} \in ITE {\text{ OR }} {\text{x}} \in IPT} \right\} {\text{ AND }} \{ {\text{x}} \in WSS {\text{ OR }} {\text{x}} \in WHI} \right\} {\text{ AND }} \left\{ {{\text{x}} \in LP {\text{ OR }} {\text{x}} \in WS} \right\}\}$$.

### Definition 3:

We define as the set of *precarious workers—level 3* the set of individuals:

$$P_{3} = \left\{ {{\text{x}}/{\text{x}}:\left\{ {{\text{x}} \in ITE {\text{ OR }} {\text{x}} \in IPT} \right\} {\text{ AND }} \{ {\text{x}} \in WSS {\text{ OR }} {\text{x}} \in WHI} \right\} {\text{ AND }} \left\{ {{\text{x}} \in LP {\text{ AND }} {\text{ x}} \in WS} \right\}\}.$$

The fourth level of precarious workers are those in involuntary temporary work with a contract that lasts less than three months or in involuntary part-time, without social security or health insurance, low-paid with no other sources apart from their work. Thus:

$$P_{4} = \left\{ {{\text{x}}/{\text{x}}:{ }\left\{ {{\text{x}} \in ITE {\text{ AND }} {\text{x}} \in LT3} \right\} {\text{ OR }} {\text{x}} \in IPT\} {\text{ AND }} \{ {\text{x}} \in WSS {\text{ OR }} {\text{x}} \in WHI} \right\} {\text{ AND }}\, \left\{ {{\text{x}} \in LP {\text{ AND }} {\text{x}} \in WS} \right\}\}.$$

## Results and discussion

The methodology described in the previous section is implemented for the case of the Greece with the use of the latest at the time EU-LFS data, i.e., for the year 2018. This analysis yields that from those respondents that are employed, 9.2% are part-time, 11.3% are temporary and 0.9% have a contract with duration less than three months. In addition, 2.3% have no health insurance, 2.4% do not have any social security and 9.5% are low-paid, i.e., they have an income which does not exceeds the amount of 560 euros, which equals the 2/3 of the median. Moreover, 0.4% of those in temporary employment are involuntary working with a fixed contract and 6.2% of part-time workers could not find a full-time job.

The criterion described in Definition 1 for precarious workers at level 1 is applied to the data set and trace 39.379 individuals that can be characterised as being in a weak precarious level. Thus, the percentage of employed individuals that belong to precarity level 1 is equal to 27.9%. Apart from providing a concrete measurement for this type of employees, the proposed methodology delivers a way of diving into the specific socio-demographic characteristics of each group.

**Fig. 7:**
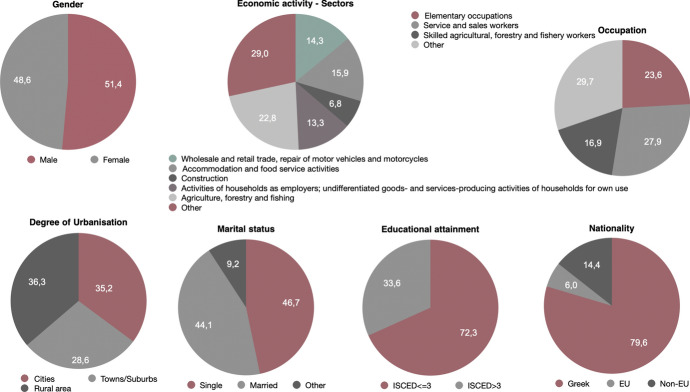
Main socio-demographic characteristics of precarious workers (level 1 – weak precarity) in Greece, EU-LFS, 2018

The analysis yields (Fig. 7) that precarious workers in the first level are almost equally divided to men (51.4%) and women (48.6%). Moreover, *Agriculture, forestry and fishing* is the sector that exhibits the highest percentage of precarious employment (22.8%), followed by the sector of *Accommodation and food service activities* (15.9%) and *Wholesale and retail trade; repair of motor vehicles and motorcycles* (14.3%). As far as the occupation is concerned, 27.9% of precarious employees are *service and sales workers*, or engaged in *elementary occupations* (23.6%), such as cleaners and helpers, and *Skilled agricultural, forestry and fishery workers* (16.9%). In relation to the degree of urbanisation of the area of residence of these workers there seems to be balanced between those living in *rural areas (thinly populated areas)* (36.3%) and in *cities (densely populated areas)* where the percentage is equal to 35.2%. The percentage of those living in *towns and suburbs (intermediate density area)* is equal to 28.6%. Approximately an equal percentage is found to be *single* (46.7%) and *married* (44.1%). When the educational attainment of respondents is examined, it is found that 72.3% is low educated (ISCED level lower or equal to 3, which means that they achieved no more than secondary education). It is important to note that 79.6% are Greek citizens, 6.0% are from another EU country and 14.4% are from a non-EU country, demonstrating that non-Greeks are relatively more than their share in the general population, since according to the latest census data the percentage of citizens having Greek citizenship is approximately 92%. The majority of precarious workers is concentrated in the region of Attica (23.7%), followed by Thessaly in central Greece which is the most significant region in terms of agricultural production (20.7%) and Central Macedonia where the second largest city in the country is located (11.6%).

**Fig. 8:**
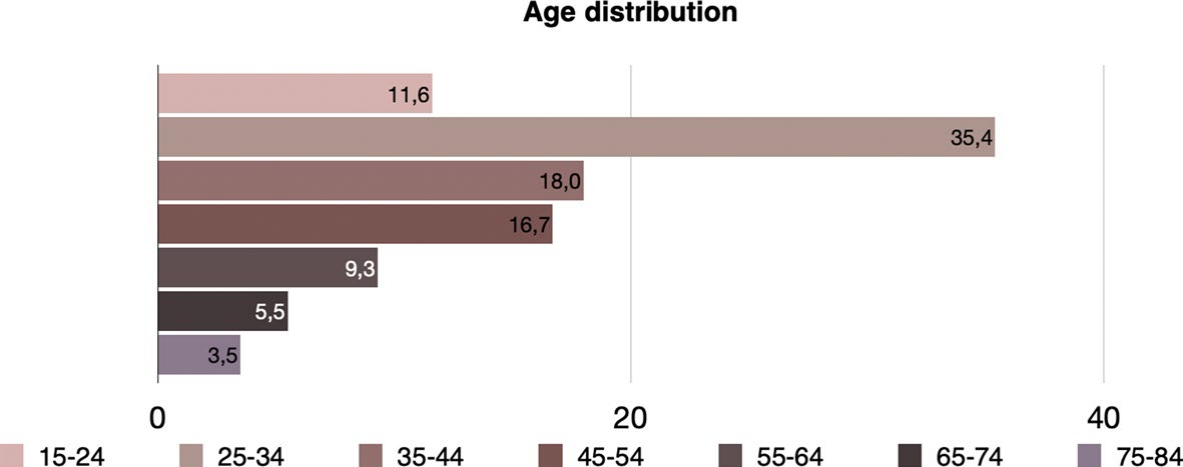
Age distribution of precarious workers (level 1) in Greece, EU-LFS, 2018

Additionally, the mean age is equal to 35.74 (minimum value is 17 and maximum is 66). The distribution of age is presented in Fig. 8. It is apparent that precarious workers at this level are young, something that adheres with the hypothesis that young individuals are much more likely than older ones to be in precarious work. The total income varies between 30 and 1000 euros, with the mean income being 364.39 euros.

**Fig. 9:**
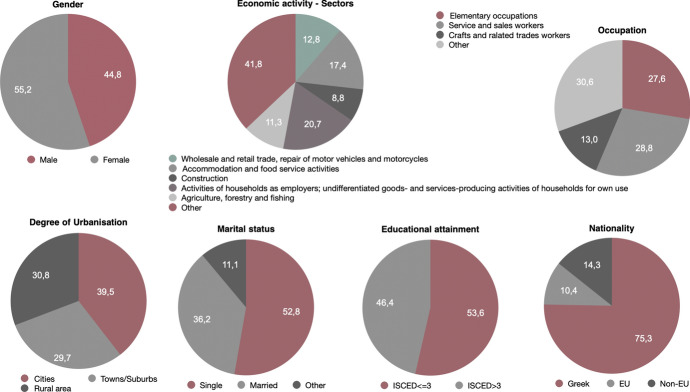
Main socio-demographic characteristics of precarious workers (level 2) in Greece, EU-LFS, 2018

As already mentioned (Sect. 3), if the criteria become stricter one can identify different levels of precarity. Taking for example the set described in Definition 2, i.e., precarious workers at level 2, thus replacing temporary and part-time employment with involuntary temporary and involuntary part-time employment the result would be 20.874 individuals, almost half of what is detected in level 1. Therefore, the percentage of employed respondents that belong to precarity level 2 is equal to 14.3%. The socio-demographic characteristics are altered (Fig. 9). A gender-based difference is detected with the percentage of women in this subset being equal to 55.2%. Interestingly, more individuals are concentrated in cities (39.5%), a higher percentage is single (52.8%), and they are better educated than those identified earlier (53.6% are low educated compared to 72.3% at level 1). As far as occupation is concerned, the sectors having the highest percentages of precarity do not change, i.e., the highest percentages are found in *service and sales workers* and *elementary occupations* with similar percentages (28.8% and 27.6%, respectively). Moreover, a shift from the Greek towards the EU population occurs. The respective percentages of precarious workers at level 1 are Greek (79.6%) and EU nationals (6%), where the ones at level 2 are 75.3% Greek and 10.4% from another EU country. A divergence also occurs in the sectors with *Activities of households as employers; undifferentiated goods- and services-producing activities of households for own use* having the highest share in precarity (20.7%), followed by *Accommodation and food service activities* (17.4%). The sector with the highest percentage (Activities of households as employers) includes home teaching (private lessons) provided by ‘free-lancers’ holding at least a bachelor’s degree, which most likely explains the increased educational status amongst levels of precarity.

**Fig. 10:**
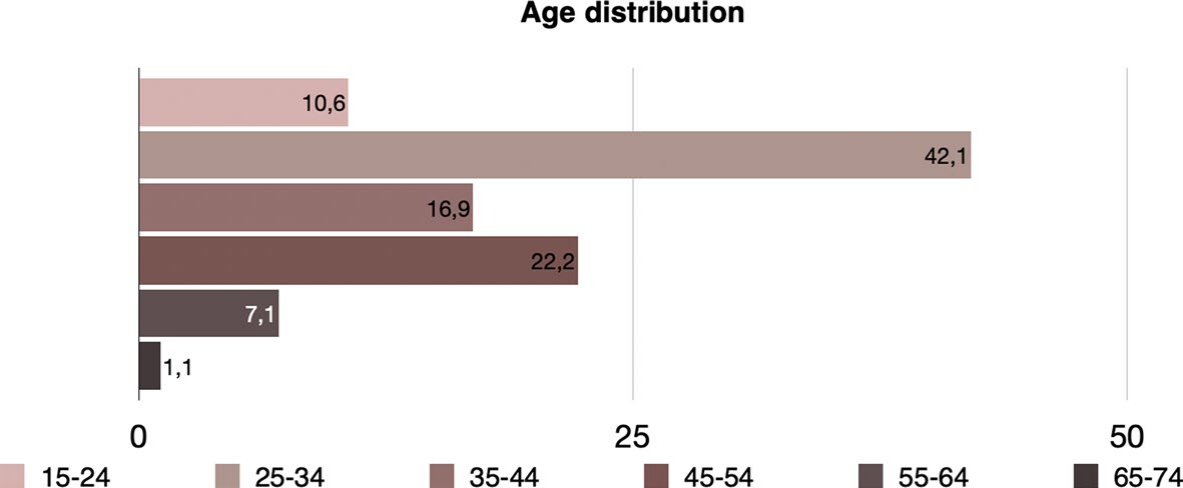
Age distribution of precarious workers (level 2) in Greece, EU-LFS, 2018

A difference in the age classes is also present. Figure 10 presents the age distribution in this case, where it is apparent that in this case almost 53% are younger than 35 years old.

**Fig. 11:**
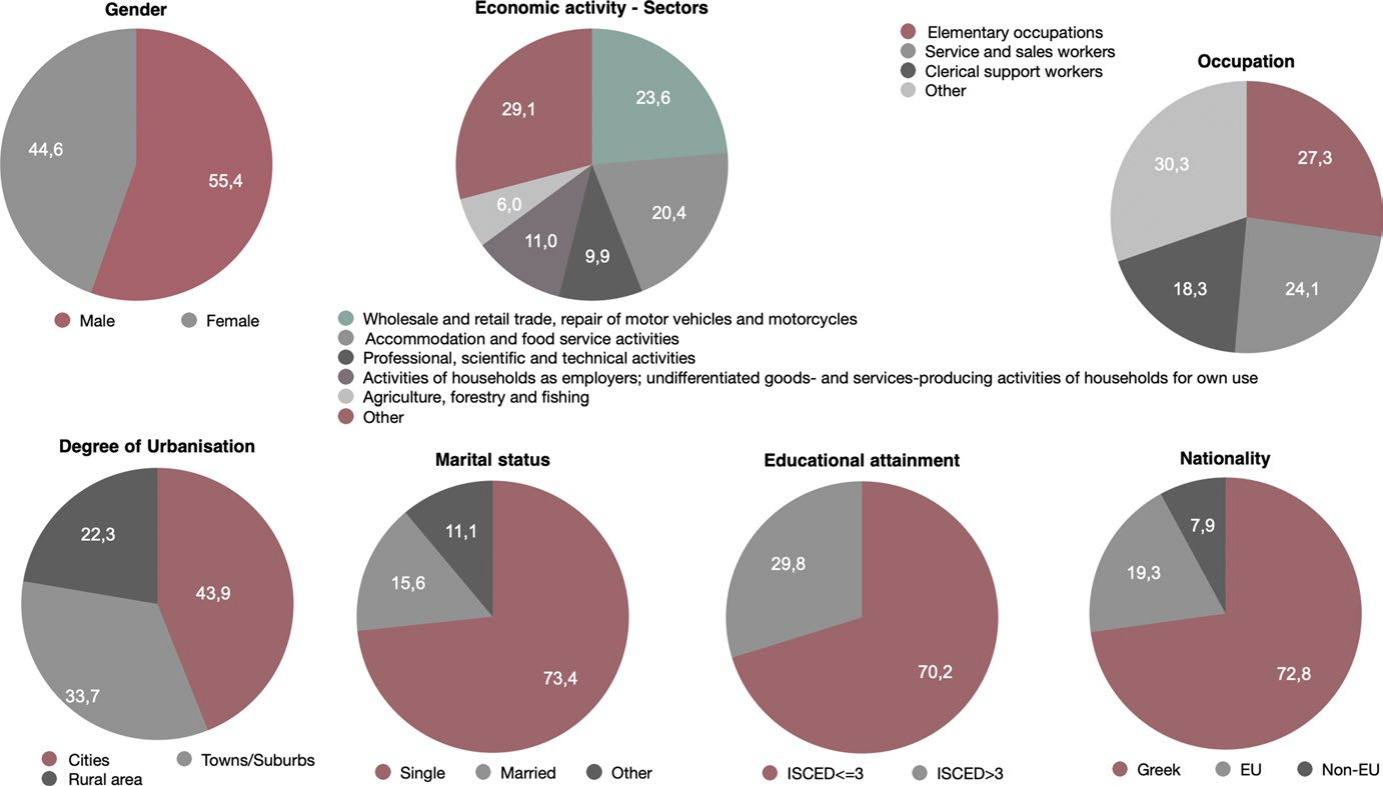
Main socio-demographic characteristics of precarious workers (level 3) in Greece, EU-LFS, 2018

**Fig. 12:**
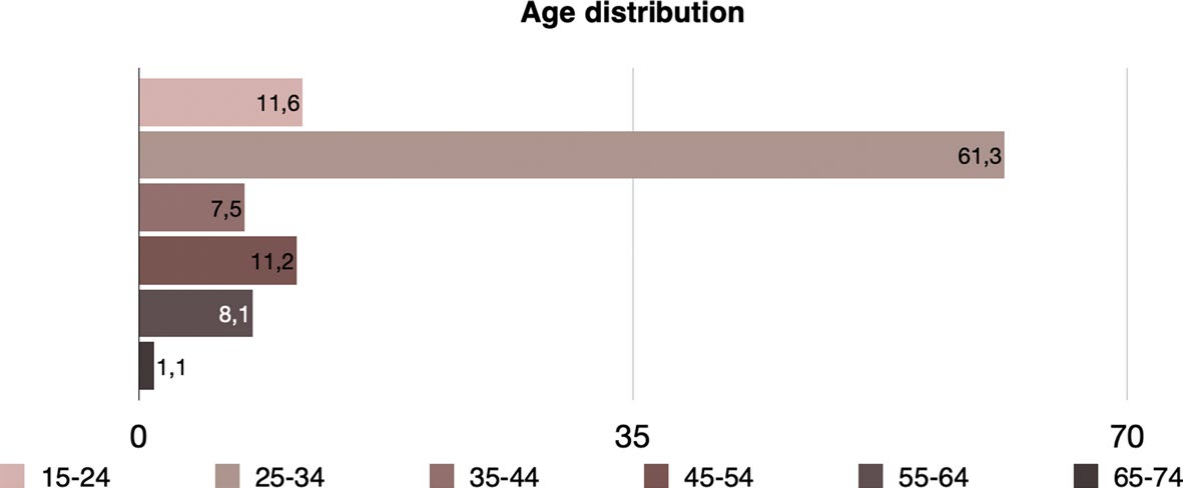
Age distribution of precarious workers (level 3) in Greece, EU-LFS, 2018

A subset that identifies even more precarious individuals (level 3) uses a stricter criterion, detecting individuals that are involuntary temporary or involuntary part-time, without social security or health insurance, low-paid that moreover have no other sources apart from their work. This results in a smaller number of precarious workers (6.134) whose socio-demographic characteristics are exhibited in Fig. 11. We can therefore conclude that the percentage of precarious workers in level 3 is equal to 4.3%. The percentage of males is now higher (55.4%) than females (44.6%). *Elementary occupations* such as helpers and cleaners are the ones presenting the highest percentage (27.3%), followed by *Service and sales workers* (24.1%). An even larger percent is found in cities (43.9% in cities, 33.7% in towns and suburbs and 22.3% in rural areas), 73.4% are single, 70.2% are low educated (up to lower secondary education). Moreover, 72.8% are Greek, but a higher percentage, equal to 19.3% corresponds to EU nationals (7.9% are non-EU). Apparently, this group is even younger in age, with 72.9% being younger than 35 years old (Fig. 12).

**Fig. 13:**
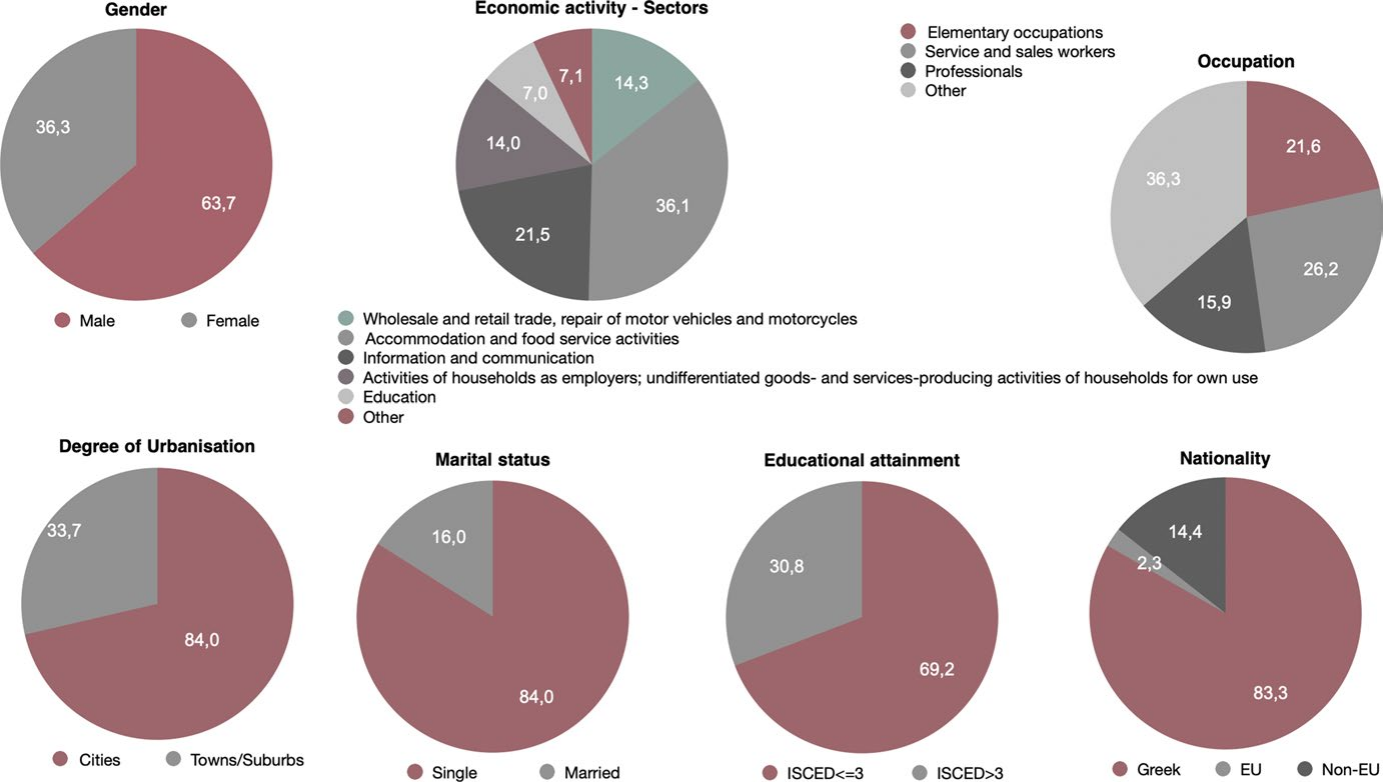
Main socio-demographic characteristics of precarious workers (level 4 – strong precarity) in Greece, EU-LFS, 2018

The fourth level of precarious workers are those in involuntary temporary work with a contract of less than three months duration or in involuntary part-time, without social security or health insurance, low-paid with no other sources apart from their work. The analysis gives 1.099 individuals, and the corresponding percentage is equal to 0.8%. These individuals are mainly male (63.7%) (Fig. 13). Regarding occupation, the sectors having the highest percentages of precarity are the same, i.e., *service and sales workers* and *elementary occupations* with similar percentages (26.2% and 21.6%, respectively), with an increase in the *professionals* (15.9%). Moreover, 84% are single, 69.2% have completed lower than secondary education and 14.4% are non-EU nationals (2.3% EU). It is also clear that 36.1% is engaged in *Accommodation and food service activities*, but a quite high percentage is found 21.5% in Information and communication. The mean age decreases to 27.58, whilst the mean income drops to 262 euros.

**Fig. 14:**
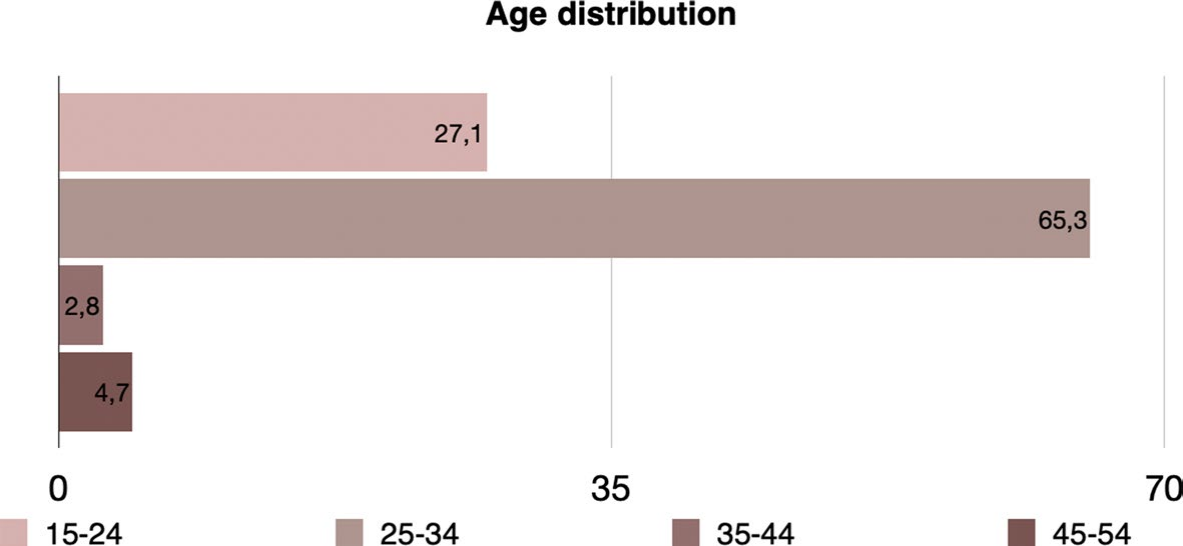
Age distribution of precarious workers (level 4) in Greece, EU-LFS, 2018

Now, to explain how the study, based on its results, is situated within the broader research landscape and to highlight the significance of the findings for advancing one’s understanding of precarious employment, we can point out that the study draws on theoretical frameworks concerning the conceptualisation of precarious employment introduced in Olsthoorn (2014), Kreshpaj et al. (2020) and Gunn et al. (2021) and support them as they prove the feasibility of the multi-dimensional construct. The implication this has for understanding precarity is significant, since a technique to measure precarity in a comparable manner is provided. Since the method introduced is novel it cannot directly be compared to previous empirical investigations on precarity. However, the results add to and confirm previous research concerning the socio-demographic characteristics of precarious workers and abides by EUROSTAT’s measurements of distinct characteristics for Greece. Similarly, a study implemented for the European Parliament (2016) that examined the development and trends of precarious employment in Europe based on the two analytical axes of employment relations and individual risk of precarity with a conceptual link to quality of work, concluded that amongst the most disadvantaged ones, are young individuals and those with lower education, key characteristics of the fourth level of precarity identified in this study. Other European studies have also reached to the same conclusion about the demographic characteristics of the most at-risk individuals (Buckingham et al. 2020; Kretsos 2010). Comparing the methodological approach of the current study to previous studies on precarity, its strengths are: (a) it captures all three dimensions agreed in the literature as important; (b) the sampling strategy of the EU-LFS allows for generalisation of the results and can produce comparable data across European countries and within. This has also significant implications for the validity and reliability of the results compared to other studies that use for example quota sampling (e.g., the European Barometer of Precariousness and Poverty Survey); (c) an innovation of the approach is that it distinguishes levels of precarity from the periphery to the epicentre (Fig. 14).

## Conclusions and further research

**Fig. 15:**
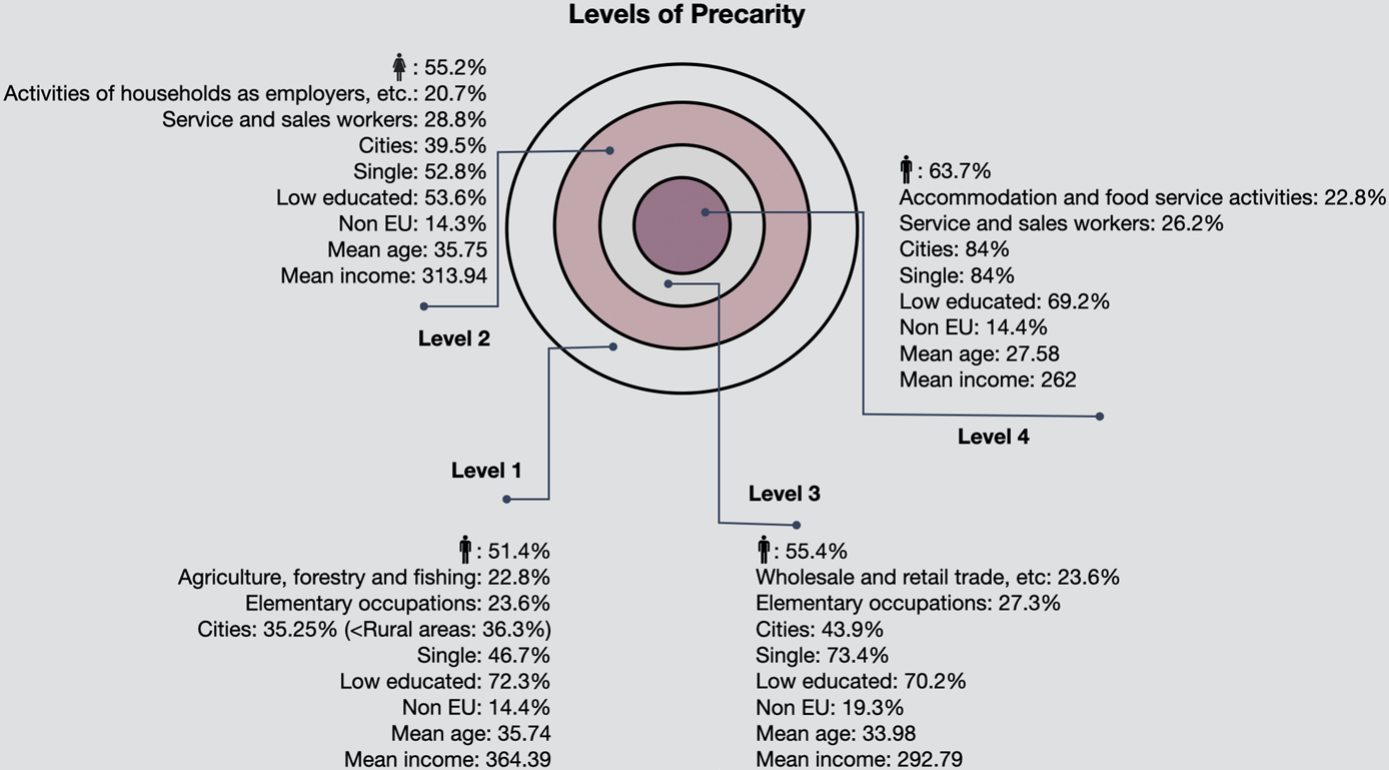
Descriptives of precarious workers per level of precarity in Greece, EU-LFS, 2018

In this paper, a set theory solution to measuring and ranking precarity is proposed. The analysis uses variables that are related to the three decisive factors associated with precarity, i.e., insufficient resources, insecurity, and unsupportive entitlements, based on Olsthoorn’s (2014) conceptualisation. These variables are all included in the EU-LFS questionnaire, and therefore the paper provides a methodology for capturing different levels of precarity amongst employed individuals in Europe. The stricter the criteria imposed on the selection, i.e., replacing logical ORs with logical ANDs, the more precarious the workers are. Moreover, the proposed methodology enables quantification of precarious workers and the examination of their socio-demographic characteristics. It is therefore apparent from the analysis performed, summarised in Fig. 15, that as precarity becomes stronger the mean age decreases, from 35.74 in the first level of weaker precarity to 27.58 in the last level where precarity is stronger. Simultaneously, the mean total monthly income decreases accordingly, dropping from 364.30 euros to 262 euros. Most workers belonging to core precarity (level 4) live in cities (84%) and are slightly better educated than those belonging to the outer level of precarity (level 1). The occupations that exhibit the highest percentages in each level, are *elementary occupations* (level 1 and 3) and *service and sales workers* (level 2 and 4). However, the sectors that exhibit the highest percentages differ in each level of precarity, being *Agriculture, forestry and fishing* in level 1, *Activities of households as employers; undifferentiated goods- and services-producing activities of households for own use*, in level 2, *Wholesale and retail trade; repair of motor vehicles and motorcycles*, in level 3 and *Accommodation and food service activities* in level 4 (Fig. 15). The percentages of non-EU nationals remain approximately the same in levels 1, 2 and 4, but interestingly is different in level 3 with a higher percentage on EU nationals. Other levels can be identified by imposing different criteria. For example, if we choose individuals that are involuntary part-time and either involuntarily working temporarily or with a short contract (less than three months) without social security or health insurance, low-paid with no other sources apart from their work we find only 131 individuals, mainly females (70.2%), all in *elementary occupations*, married (70.2%), all very low educated (ISCED level 1 or 2), that all come from a non-EU country and are aged up to 35.

These results have significant implications for both theory and practice. As far as the theoretical implications are concerned, the proposed method delivers a set theory solution to defining precarity but also identifying different levels of precarious workers. This clearly adds to the current discussion on precarity and its conceptualisation, which is central to the entire discipline of sociology of work and labour statistics. This paper, having as a starting point Olsthoorn (2014) conceptualization of precarity carefully systematizes it using set theory. To our knowledge, this is the first time that different levels of precarity are defined and linked with specific well-defined collections of elements. The need to address this complex phenomenon is widely recognised, given its multifaceted nature (Eurofound 2018). Moreover, as earlier stated, the European Parliament recognised a definition of precarious employment as “employment which does not comply with EU, international and national standards and laws and/or does not provide sufficient resources for a decent life or adequate social protection”. The proposed methodology is fully compliant with this definition as it defines precarious workers as those with insufficient resources, and/or inadequate social resources (health insurance and/or social security) and features implying different levels of job insecurity. Another strength of the suggested methodology is the fact that it provides a way of selecting individuals that belong to different levels of precarity and then thoroughly examining their socio-demographic characteristics to extract knowledge concerning these categories.

From a practical point of view, the study provides a clear way to identify different levels of precarious workers in Europe using raw data drawn from a widely used, large scale sample survey, the EU-LFS. Common definitions, questions and variables make the suggested methodology replicable to the thirty-five participating countries. It is also important to mention that analysing data from 2018 can be used for baseline comparisons to help us understand the impact of the COVID-19 pandemic on precarity and how the pandemic has exacerbated the phenomenon. Relevant studies show that the COVID-19 crisis highlighted a growing precarity in employment and had significant effects on workers’ well-being (Donato et al. 2022; Pun et al 2022; Wu 2022 amongst others). As far as future directions are concerned further work would include the application of this methodology to other EU member datasets to produce cross-national comparisons and the EU-LFS dataset for Greece for the subsequent years. The possibility of examining an experts’ knowledge system to suggest a weighting scheme for constructing the levels of precarity could also be an aspect of future work. First steps towards this direction have already been made (Stamou et al. 2022a, 2022b).

The present analysis contributes to a more concrete situatedness of precarity in labour market and within economic activities. In the relevant theoretical literature, precarity is often linked to aspects of cognitive capitalism, such as creative industries or other forms of immaterial labour, which is defined as the production of commodities that are constituted by their cultural, emotional, creative, or intellectual content, and where labour can be understood as the process in which work becomes mainly subjective and communicative (O’Doherty and Willmott 2001). Evidence from the Greek case study shows that precarity emerges in a variety of sectors, which are not necessarily linked to cognitive aspects of the economy. Moreover, precarious workers cannot be considered as a homogeneous group with common characteristics and potential.aspirations.

Due to the use of probabilistic sample design in the EU-LFS, reliable inferencing about the entire population is allowed. We could therefore conclude that the percentages of employed individuals that belong to different levels of precarity in Greece are for level 1 (weak precarity) 27.9%, for level 2, 14.3%, for level 3, 4.3% and for the 4^th^ level of strong precarity 0.8%. The proposed 4-level typology aims at capturing what is described in Norbäck and Styhre (2021) as *the spectrum of precarity* or *precarity to different degrees*. Given the breadth of possibilities in defining precarious employment, we have attempted to limit the scope to four levels of precarity capturing both ends (weak and strong precarity) along with two intermediate levels. The distinct, decreasing percentages from one level to the other confirm this choice.

## Supplementary Information

Below is the link to the electronic supplementary material.

Supplementary file1 (DOCX 32 kb)

## Data Availability

The data that support the findings of this study are available from the National Statistical Authority of Greece (ELSTAT) or EUROSTAT. Data were used under licence for the current study, are not publicly available but can be reached via an application to either ELSTAT (https://www.statistics.gr/en/scientific_provision_data) or EUROSTAT’s micro-data access support (https://ec.europa.eu/eurostat/web/microdata/european-union-labour-force-survey).
