# Supplementary material for: Measuring the unmeasurable: defining and rating precarity with the aid of EU-LFS data
Source: SN Soc Sci. 2023 Mar 22;3(4):67. doi: 10.1007/s43545-023-00651-5 (PMC10032629; doi:10.1007/s43545-023-00651-5)
Supplement: Supplementary file 1 — Supplementary file1 (DOCX 32 kb) [file 43545_2023_651_MOESM1_ESM.docx]

**Appendix A: Sets and their basic notation**

Set theory is a branch of mathematics and more precisely of Mathematical Logic dedicated to the study of collections of objects, their properties, and the relationship between them. A set $A$ is a collection of all elements $x$ that satisfy a given property, i.e., a set is a collection of well-defined objects. It is therefore possible to examine whether an object is an element of $A$, i.e., if the object belongs to the set, by examining whether the object has the respective property. The property is usually denoted by $p(x)$, and the set by $A=\{x/x: p(x)\}$, meaning that the set $A$ is a collection of all $x$’s that have the property $p(x)$.

**Basic notation**

| $\emptyset$ | The empty set. The empty set contains no elements whatsoever. |
| --- | --- |
| $x\in A$ | The object $x$ belongs to a set $A$ ($x$is an element of $A$). |
| $x\notin A$ | $x$ does not belong to $A$ ($x$is not an element of $A$). |
| $A\cup B$ | The union of two sets $A$ and $B$ include elements that belong to set A or set B. It is true that $A\cup B=\{x/x: x\in A \vert x\in B\}$. |
| $A\cap B$ | The intersection of two sets $A$ and $B$ include elements that belong both to set A and set B. Therefore, we can write $A\bigcap B=\{x/x: x\in A and x\in B\}$. |
| $A\subseteq B$ | Every element of set $A$ is an element of $B$. |

**Appendix B: The European Union Labour Force Survey and replication of the method to other national data bases**

**Overview of the European Union Labour Force Survey**

The EU-LFS is the largest European sample survey offering quarterly and annual statistics on labour market participation and inactivity of individuals over 15 years old. It is a household survey covering residents in private households in thirty-five participating countries providing EUROSTAT -responsible for centrally processing the data- with valuable information. Data are drawn from national labour force surveys conducted by the National Statistical Institutes (NSIs) in accordance with Regulation (EU) 2019/1700. EUROSTAT oversees the implementation of the Regulation, aids the NSIs and recommends harmonised concepts and methodologies. Each NSI is authorised for designing national questionnaires, drawing the sample, conducting interviews, and returning the results to EUROSTAT in agreement with a common coding scheme based on the Commission Implementing Regulation (EU) 2019/2240. The NSIs apply the design which fits the most their quality standards and needs, taking into consideration the national specificities among others, however all participating countries are obligated to use a probability sampling design (random sampling). More specifically, all countries except Lithuania, Luxembourg, Malta, and Iceland use a stratified random sampling method. The stratification is mainly done on geographical areas, but the degree of urbanisation is also a frequently used stratification variable. In general, the most common sample design for the EU-LFS is the stratified two-stage cluster sampling followed by the stratified one-stage cluster sampling. The systematic random sampling (without stratification) is only applied in Malta. The use of probabilistic sample design permits reliable inferencing about the entire population and the quantification of estimates’ precision based on variance estimation.

To monitor the quality of the EU Labour Force Survey (EU-LFS) there are specific reports that are conducted on an annual basis: the main characteristics of the national sample surveys report and the quality report. The former allows users to interpret the EU-LFS outcomes by presenting evidence concerning the technical characteristics of the Labour Force Surveys conducted by the NSIs in each participating country. The latter helps users assess the quality of the statistics drawn from the EU-LFS, as it provides a summary of the key quality indicators, namely: relevance, accuracy, accessibility and clarity, timeliness and punctuality, comparability, and coherence. Most countries adjust for non-response either directly during the weighting process or in a preliminary step. Moreover, as is the case in most sample surveys, EU-LFS faces the problem of imperfect frames leading to coverage errors, i.e., discrepancies between the survey population and the target population. For example, in the EU-LFS 2019 wave, nine countries report both under-coverage (the frame population does not include all units of the target population) and over-coverage (the frame population includes units that do not belong to the target population). On the other hand, Italy is the only country reporting misclassification (some units of the frame population that are part of the target population are misclassified). The reader can consult the EU-LFS Quality Report (2022) for a detailed description of coverage errors, item and unit non-response in the EU-LFS and the evolution of the respective rates from 2015 and onwards. Exhaustive information is also provided for the procedures followed by the NSIs to reduce measurement errors.

The EU-LFS serves as a major source of information for other important European statistics, due to the diversity of the questions posed to respondents and the sample size. Each quarter, more than 1.7 million interviews are conducted, with larger countries having larger samples (such as Germany, Spain and Italy) and countries with smaller population size smaller (like Malta and Iceland). Apart from having large representative samples, EU-LFS encompasses an additional strong element, which is the comparability across countries and over time. To ensure that this is possible the EU-LFS uses common concepts and definitions, follows International Labour Organization (ILO) guidelines, uses the same classifications (such as ISCO, ISCED, NUTS), and records the same set of variables in each country. More specifically, a common framework regulation (Council Regulation (EC) No 577/98), common variable definitions (Commission Regulation (EC) No 377/2008), common explanatory notes (EU Labour Force Survey Explanatory Notes) and a common directive concerning the definition of unemployment and the twelve principles of questionnaire construction (Commission Regulation (EC) No 1897/2000) guarantee the comparability of estimations amongst participating countries. The coherence of employment and unemployment rates for the EU-LFS and National Accounts is also presented in the EU-LFS Quality Report (2022). The employment estimates based on EU-LFS data usually lie slightly under the evaluations of employment estimated by National Accounts, particularly in countries with a significant percentage of an irregular economy. In some countries unemployment figures based on the EU-LFS exceed the estimated provided by registers, while in other the opposite is true. The main sources of divergencies between unemployment rates estimated through the EU-LFS and those derived from registers are the different definitions and measurement methods. For example, the EU-LFS makes use of the ILO definition for unemployment, which means that a person is considered unemployed if he/she satisfies the following conditions: he/she is not employed during the reference week, is available to start work within the two weeks following the reference week and is actively seeking work in the four weeks preceding the reference week or having already found a job to start within the next three months. However, it is important to note that all countries use the same definitions and measurement methods that make comparisons between participating countries possible.

**Replication of the method to other European countries**

Most variables used in the analysis presented in the paper are core variables that exist in all questionnaires of participating countries. Core variables are transmitted by the NSIs to EUROSTAT according to the latest Commission regulation on codification. These variables are TEMP, TEMPREAS, TEMPDUR, FTPT, FTPTREAS and they can be used to measure temporary employment, reason for having a job/work with limited contract, the duration of the contract, part-time employment, and reason for having part-time employment. Moreover, it is compulsory since 2009 for the NSIs to include a variable called INCDECIL, which measures the monthly take home pay from main job in deciles. Therefore, workers having a monthly income less than the 3^rd^ decile (INCDECIL value $\leq$ 3) can sufficiently proxy the $\frac{2}{3}\cdot$median (the 3^rd^ decile equals the 30-th percentile and the $\frac{2}{3}$ of the median the 33-th percentile). In addition, a combination of the variables EXIST2J (existence of second job) and household information (e.g., HHWKSTAT, working status of adults living in the same households) can produce information about other sources of income. Finally, the two variables used in this analysis concerning social security and health insurance are not core variables, but the choice for inclusion was made by the Greek NSI (ELSTAT). The purpose of these variables is to capture differences amongst respondents concerning social security and health insurance, allowing them to choose “I have no social security” and/or “I have no health insurance”. We note here that this is possible in the case of Greece. However, for all participating countries in the EU LFS, it is possible to capture social benefits recipients, through a core variable called REGISTER, by selecting respondents that have (or have not) received any benefits or assistance. On top of that, in each country’s questionnaire there exist other specific questions adjusted to the national context that could be used. For example, in the UK one can find a whole section, called the Benefit Entitlements section, with eleven questions asking about State benefits and Tax credits that the respondent may be receiving. Similar variables are included in the other participating countries questionnaires.

**Appendix C: Categories of insecure employment, unsupportive entitlements, and insufficient resources**

**Categories of insecure employment**

- Full-time employed, but working involuntarily temporarily, with a limited contract of duration greater than three months,
- Full-time, but working involuntarily temporarily, with a limited contract of duration less than three months,
- Full-time, but working voluntarily temporarily, with a limited contract of duration greater than three months,
- Full-time, but working voluntarily temporarily, with a limited contract of duration less than three months,
- Voluntarily part-time, also working involuntarily temporarily, with a limited contract of duration less than three months,
- Voluntarily part-time, also working involuntarily temporarily, with a limited contract of duration greater than three months,
- Involuntarily part-time, involuntarily temporarily, with a limited contract of duration greater than three months,
- Involuntarily part-time, involuntarily temporarily, with a limited contract of duration less than three months,
- Involuntarily part-time, voluntarily temporarily, with a limited contract of duration less than three months,
- Involuntarily part-time, voluntarily temporarily, with a limited contract of duration greater than three months,
- Voluntarily part-time, voluntarily temporarily, with a limited contract of duration greater than three months,
- Voluntarily part-time, voluntarily temporarily, with a limited contract of duration less than three months,
- Voluntarily part-time with a permanent contract, and
- Involuntarily part-time with a permanent contract.

**Categories of unsupportive entitlements**

- With social security, but without health insurance.
- With no social security and no health insurance.
- With health insurance, but without social security.

**Categories of insufficient resources**

- Low-paid (income< $\frac{2}{3}\cdot$median) with other sources.
- Low-paid (income< $\frac{2}{3}\cdot$median) without other sources.
- Income> $\frac{2}{3}\cdot$median without other sources.
